# Editorial: The Natural World as a Resource for Learning and Development: From Schoolyards to Wilderness

**DOI:** 10.3389/fpsyg.2019.01763

**Published:** 2019-07-31

**Authors:** Ming Kuo, Catherine Jordan

**Affiliations:** ^1^Landscape and Human Health Laboratory, Department of Natural Resources and Environmental Sciences, University of Illinois at Urbana-Champaign, Urbana, IL, United States; ^2^Department of Pediatrics, Medical School, University of Minnesota, Minneapolis, MN, United States; ^3^Children and Nature Network, Minneapolis, MN, United States

**Keywords:** nature, natural environments, learning, development, education

Children are increasingly losing contact with nature. Many live in highly developed settings with relatively few natural elements or views; others have access to some forms of nature but spend the vast majority of their time indoors. What are the consequences of this shift for children—their academic achievement, what they know and don't know, their values and abilities, and who they become? And what are the consequences for the rest of us?

Increasing evidence suggests that the natural world may be a powerful resource for learning and development. “Contact with nature,” ranging from wilderness vacations to catching frogs in a drainage ditch to doing homework with a view of trees, is increasingly tied to positive outcomes. These discoveries raise the tantalizing potential of identifying low-cost ways to address major societal challenges: boosting academic achievement, reducing the achievement gaps between different ethnic and socioeconomic groups, and countering the rise in various mental and physical disorders.

This Research Topic expands our understanding of the natural world as a resource for learning and development. These 12 articles:

- **Capture the current state of the evidence in this area**, revealing far stronger converging evidence for the positive role of nature in learning and development than previously realized and identifying a set of mechanisms underlying the relationship between nature and learning (Kuo et al.).
- **Expand our understanding of the populations nature-based learning (NBL) can serve**—while much of the previous research in this area has focused on relatively well-off, mainstream elementary school populations, the work here extends the benefits of nature to preschool children (Agostini et al.); children from low-income and/or minority neighborhoods (Bates et al.; Kuo et al.; Kuo et al.); children with emotional, cognitive, and behavioral disabilities (Szczytko et al.); and children around the globe, including from the United States, Italy, and Denmark.

- **Expand our understanding of the many ways in which children might experience nature**. At school, children might engage in project-based learning about a living wall in their classroom (McCullough et al.), have lessons outdoors (Barfod and Daugbjerg; Kuo et al.; Szczytko et al.), or have recess in a green outdoor area (Amicone et al.). Away from school, experiences like “being in solitude in nature” or “cohabiting with a wild animal” might contribute to healthy development (Kahn et al.).

- **Expand our understanding of the potential benefits of time in and around nature**.
  - A handful of articles address cognitive and academic benefits of nature: recess that mentally rejuvenates (Amicone et al.); lessons that boost student engagement with the current lesson (Szczytko et al.) and subsequent lessons (Kuo et al.); schoolyards that may boost standardized test performance (Kuo et al.); and green outdoor settings that seem to elicit and support child-centered, inquiry-based instructional methods (Barfod and Daugbjerg), serve as an antidote to cognitive load from technology (Schilhab et al.) and boost cognitive and language development (Agostini et al.).
  - Other articles provide evidence for physical benefits including increased physical activity (Bates et al.) and improved gross and fine motor skills, body function, and awareness of the surrounding environment (Agostini et al.).
  - Finally, several articles address social, emotional and behavioral functioning, including increases in prosocial interactions (Bates et al.), reductions in disruptive behavior (Szczytko et al.), and increased play and enhanced social and emotional development (Agostini et al.).

- **Offer a roadmap for future research**, identifying some of the most important and pressing “game-changing” research questions at this time (Jordan and Chawla).

The diversity of disciplines represented in this collection illustrates the importance of diverse backgrounds and areas of expertise in understanding the role of nature in children's learning and development. The authors' affiliations here span forest and natural resources; pediatrics; parks, recreation and tourism; teacher education; informatics; social work; environmental psychology; developmental psychology; landscape architecture; environmental education; nutrition; health promotion; and future technology, culture, and learning. Perhaps not surprisingly, the methodologies are similarly diverse and offer a rich picture of nature's impact. For example, direct observation of children's play, examination of nature-based instructional sessions, teacher interviews, cognitive testing and standardized tests each offer a unique perspective that contributes to our understanding.

The work here attests to the multiple benefits of nature contact across a variety of settings and forms of engagement. These original research studies, reviews, and conceptual pieces have implications for diverse fields, including education, teacher preparation, early childhood development, design and planning, and health and mental health care, among other sectors. Taken together, this work argues for practitioners and decision makers to value nature contact as a critical resource for children's learning and development.

## Author Contributions

Both authors compiled information about the 12 articles. MK provided an outline and first draft. CJ provided additional text. Both authors revised and approved the manuscript for submission.

### Conflict of Interest Statement

The authors declare that the research was conducted in the absence of any commercial or financial relationships that could be construed as a potential conflict of interest.

